# A Simple Low-Cost Method to Prepare Lignocellulose-Based Composites for Efficient Removal of Cd(II) from Wastewater

**DOI:** 10.3390/polym11040711

**Published:** 2019-04-18

**Authors:** Yingying Wen, Yong Ji, Shifeng Zhang, Jie Zhang, Gaotang Cai

**Affiliations:** 1 College of Water Conservancy and Ecological Engineering, Nanchang Institute of Technology, Nanchang 330099, China; wyying1023@163.com (Y.W.); shifeng.zhang@bjfu.edu.cn (S.Z.); zhangjie@nit.edu.cn (J.Z.); nitkyc@126.com (G.C.); 2 Beijing Key Laboratory of Wood Science and Engineering, Beijing Forestry University, Beijing 100083, China

**Keywords:** wood waste, lignocellulose composites, PEI, coating, adsorption

## Abstract

The fabrication of functional lignocellulose-based materials has drawn considerable attention because it acts as a green separation/absorption material owing to its multi-porous mesostructure. In this study, a surface functionalized lignocellulose-based adsorbent for the highly efficient capture of Cd(II) ions was prepared through facile in situ co-deposition of wood waste-derived saw powder (SP) in the presence of tannic acid (TA) and aminopropyltriethoxysilane (APTES) mixed aqueous solution. The SP was first modified using TA-APTES coating to synthesize the functional SP substrate (SP-(TA-APTES)). The SP-(TA-APTES) hybrids served as reactive platforms, which enabled further decoration with amino-rich polyethylenimine (PEI) due to the outstanding secondary reactions of the TA-APTES layer. The surface morphology of the resulting SP-(TA-APTES)-PEI (SP-TAPI) composites were investigated using Fourier-transform infrared spectroscopy (FTIR), X-ray photoelectron spectroscopy (XPS), X-ray diffraction (XRD), thermogravimetric analysis (TGA), and scanning electron microscopy (SEM). Significantly, the combined advantages of the lignocellulosic skeleton, the layer-particle structure, and the hybrid coating contributed to the enhanced adsorption capacity of Cd(II) (up to 22.66 mg/g at pH = 5.0). This removal capacity was higher than that of most reported agricultural waste-based or lignocellulose-based materials. The Cd(II) adsorption mechanism of the surface-modified SP-TAPI composites was studied in detail. These results provide new insights into the high value-added utilization of agricultural waste for water purification applications.

## 1. Introduction

Environmental contamination of the aqueous environment is an increasing global concern for human health [1,2]. The extensive release of heavy metals and organic dyes over the past decades have led to widespread water pollution [3,4,5]. Specifically, cadmium (Cd(II)) is a common high-toxic metal contaminant originating from numerous industries, such as printing, textile, and metal plating [6,7]. To date, many technologies have been developed to remove the metal ions and organic compounds from wastewater, including physical adsorption, membrane filtration, ion exchange, chemical precipitation, photocatalytic biodegradation, and electrochemical treatment [4,8]. Among the various treatment techniques, adsorption has become the major technique and has been extensively applied for the disposal of wastewater because it is low-cost, shows high efficiency, and is simple in design [9,10]. In recent years, sustainable sorbents prepared from renewable biomass such as cellulose and/or lignocellulose-based materials, chitosan, and alginate, have received increasing attention [11,12]. Among these biosorbents, lignocellulose is considered a promising and cheap biosorbent [13,14]. Compared with other resources, it is a natural lignocellulosic material composed of abundant free carboxyl and hydroxyl groups, which provide reactive sites for further surface modification [15]. In addition to its “green” advantages and biocompatibility, lignocellulose has a unique advantage for adsorption because it possesses a large specific surface area and natural porous structure [16].

Wood, a porous and multi-hierarchical lignocellulosic material, exhibits unique properties such as an intrinsic mesoporous structure, outstanding optical and mechanical properties, and excellent water transportation capacity, thereby making it the most promising candidate for wastewater treatment [17,18]. Chen et al. reported on a Pd-decorated 3D wood membrane fabricated from engineered natural wood for the removal of methylene blue (MB) from aqueous solutions; a high removal efficiency was achieved (≥99.8) [19]. Vitas et al. found that carboxylic acid functionalized wood could be used for the removal of copper(II) from water [20]. To date, most studies have focused on deriving wood-based sorbents from solid wood and nanocelluloses extracted from wood, and only limited efforts have been devoted to the direct use of wood waste as a lignocellulosic source [12,21]. Furthermore, most processing procedures involve the use of strong acids and have high-cost and energy waste, which impedes sustainable development [22,23]. Thus, our goal is to use wood waste for the treatment of contaminated water and ultimately achieve the value-added utilization of natural resources using a “green” and low-cost strategy.

Practical applications of wood waste-type sorbents have been largely hindered by insufficient Cd(II)-binding sites or abilities on the surfaces [19,24]. It is highly desirable to develop a facile and efficient approach to prepare functional surfaces for adsorption on the lignocellulose substrate. In this study, we demonstrated for the first time that the use of saw powder (SP) substrates (derived from the processing of wood-based products) yields a naturally low-cost sorbent with highly reactive amino (−NH_2_) groups on the surfaces. First, SP substrates with a hierarchical structure were coated with an adhesive film by immersion in an aqueous solution of tannic acid (TA) and γ-aminopropyltriethoxysilane (APTES) via a pyrogallol-amino covalent bridge. The TA molecules have relatively strong surface-independent adhesion and exhibit a good chelation capability to metal ions [25,26]. Moreover, they possess abundant catechol and pyrogallol units, which serve as versatile reactive platforms, thus facilitating the adherence of the amino-terminal polymer on the inorganic and organic substrates [27,28]. Thus, the amino-rich polyethylenimine (PEI) molecules were covalently grafted on the as-obtained rough TA-APTES-coated SP via a Michael addition or Schiff base reaction between the free pyrogallol moieties and amine groups. The co-deposited TA-APTES coating created a hierarchical structure of the SP sorbent and improved its specific surface, while the presence of abundant −NH_2_ groups significantly increased the adsorption capacity toward Cd(II) ions. The surface chemistry and microstructure of the prepared SP/TA-APTES/PEI (SP-TAPI) sorbent was evaluated. The effects of various environmental factors on the removal of Cd(II) by the SP-TAPI biosorbent were investigated, including the pH, adsorption time, temperature, and the initial concentration. Additionally, energy-dispersive X-ray (EDX), Fourier-transform infrared spectroscopy (FTIR), and X-ray photoelectron spectroscopy (XPS) were performed to analyze the adsorption mechanism of the composites.

## 2. Materials and Methods

### 2.1. Materials

Chinese fir (*Cunninghamia lanceolata*) SP (~80–100 mesh) from Wen’an Plywood Ltd. (Hebei, China) was used. The SP was first purified by washing with distilled water and dried in an oven at 103 ± 2 °C for 24 h. The TA, APTES, PEI (Mw = 600), tris(hydroxymethyl)aminomethane (Tris), and cadmium chloride (CdCl_2_) were purchased from Tianjin Heowns Biochem. Co., Ltd. (Tianjin, China). All chemicals were analytical grade and used as supplied.

### 2.2. Preparation of SP-TAPI Composites

The SP/TA-APTES was prepared by a two-step deposition method. First, an SP aqueous dispersion with a concentration of 0.2 wt % (500 mL) was prepared by an ultrasonic-assisted method under continuous stirring for 6 h. The pH value of the mixed solution was adjusted to 8.5 using Tris-HCl buffer solution (10 mM). Next, 0.25 g of APTES was dissolved in 50 mL of ethanol and 0.5 g of TA was added to the suspension, followed by stirring at 30 °C for 24 h. Then, the modified substrates (denoted as SP/TA-APTES) were washed repeatedly by ethanol and deionized water to remove impurities from the coating and were vacuum-dried at 50 °C for 12 h. Subsequently, 0.2 g of the as-prepared SP/TA-APTES were dispersed in deionized water, followed by adding a certain amount of PEI solution under vigorous mechanical stirring at 25 °C for 12 h. Finally, the synthesized SP-TAPI hybrids were washed several times with deionized water and vacuum-dried at 50 °C overnight.

### 2.3. Characterization

The surface morphology characterization and elemental mapping were performed using field-emission scanning electron microscopy (FE-SEM, Hitachi SU8010, JEOL, Tokyo, Japan) in combination with EDX. The phase structure of the composites was analyzed by X-ray diffraction (XRD) on a Bruker AXS D8 diffractometer (Karlsruhe, Germany) operated at a voltage of 40 kV and 40 mA with Cu Kα radiation (λ = 0.154 nm). Thermogravimetric analysis (TGA) was carried out in N_2_ atmosphere with a TGA instrument (Q50, TA Instruments, New Castle, DE, USA) from 25 to 600 °C at a heating rate of 10 °C min^−1^. The attenuated total reflectance FTIR (ATR-FTIR) spectra were obtained using a Nicolet 6700 spectrometer (Thermo Scientific, Madison, WI, USA) from 4000 and 400 cm^−1^. X-ray photoelectron spectroscopy (XPS) (Thermo Scientific, Madison, WI, USA) with Al Kα radiation (1486.6 eV) was performed to determine the cadmium form after being by the SP-TAPI absorbents.

### 2.4. Adsorption Experiments of Cd(II)

The trapping performance of the Cd(II) on the SP-TAPI was systematically evaluated by batch experiments. Briefly, 100.0 mg of SP-TAPI was introduced into the Cd(II)-containing stock solution (50 mL, initial pH at 7.0, 50 mg L^−1^) for 60 min of reaction time. The removal capacity of the wood waste-derived SP-TAPI was investigated at different initial concentrations (10–100 mg L^−1^). To evaluate the pH influence on the Cd(II) removal, the pH values of the solutions (50 mg L^−1^) were adjusted, ranging from 2 to 6 by using NaOH and HCl (0.1–1 M). In addition, 50 mg L^−1^ CdCl_2_ solutions with different pH values were used to study the effect of the solution temperature (30, 40, 60, 80 °C) and the contact time. Subsequently, the suspension was shaken at 150 rpm for 24 h to ensure adsorption equilibrium. After centrifugation, the residual Cd(II) concentration in the supernatant was measured using the inductively coupled plasma (ICP) method (Optima 8X00, Waltham, MA, USA). The removal percentage (RP) was calculated using the following equation:

(1) $$\mathrm{RP}=\frac{C_{e}-C_{0}}{C_{0}}\times 100\%$$

where $C_{0}$ and $C_{e}$ (mg L^−1^) are the initial and equilibrium concentrations of the Cd(II) ions.

## 3. Results and Discussion

### 3.1. Structural Characterization of SP-TAPI Absorbents

**Scheme 1:**
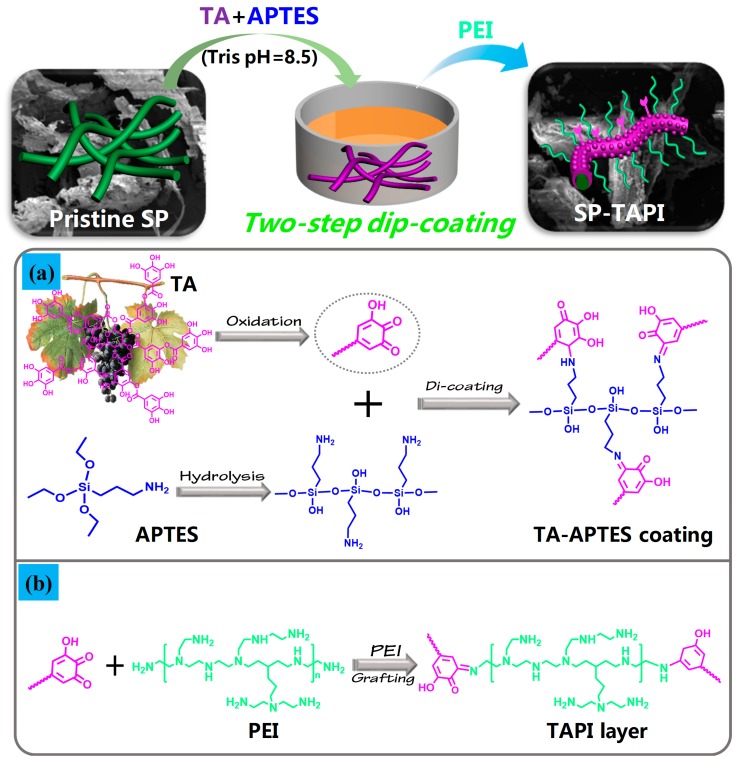
Schematic illustration of the preparation of hybrid saw power (SP) tannic acid (TA) aminopropyltriethoxysilane (APTES) polyethylenimine (PEI) composites, collectively referred to as SP-TAPI composites, and possible interaction mechanism of two-step deposition of TA, APTES and PEI.

Scheme 1 presents the formation mechanism of the two-step deposition of the TA-APTES coating and PEI hybrid on the SP substrate. Owing to the self-polymerization of the TA in the alkaline solution, the different types of generated quinone moieties reacted with APTES through a Michael addition and Schiff base reaction to form an adhesive cross-linking network, which resulted in the formation of a rough porous layer (TA-APTES) on the SP substrates [29,30]. Furthermore, it was easy to graft the amino-rich PEI onto the surface and provide sufficient Cd(II)-binding sites and abilities of the resulting SP-TAPI composites. Because the work presented here aimed to fabricate lignocellulose-based SP-TAPI composites as a sustainable absorbent, a lignocellulosic substrate of pristine SP and the (TA-APTES)-functionalized SP served as control samples.

**Figure 1:**
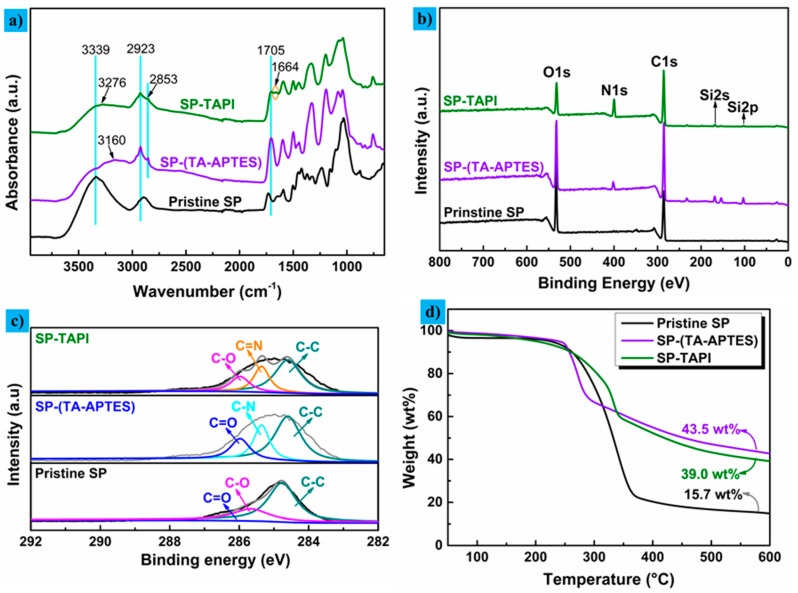
(**a**) Fourier-transform infrared spectroscopy (FTIR) spectra of pristine SP, SP-(TA-APTES), and SP-TAPI. (**b**) Wide-scan XPS spectra and (**c**) deconvolution curves of C 1s spectra of pristine SP, SP-(TA-APTES), and SP-TAPI. (**d**) Thermogravimetric analysis (TGA) curves of pristine SP, SP-(TA-APTES), and SP-TAPI.

As shown in Figure 1, FTIR and XPS analysis were used to confirm the change in functionalities during the co-deposition process. In Figure 1a, the pristine SP exhibited some of the typical peaks of lignin and polysaccharides. After the TA-APTES modification, the peak of the SP-(TA-APTES) was located at 1705 cm^−1^ and ascribed to the C=O stretching vibration, owing to the quinone structure from the catechol group in the TA molecules [31,32]. The peak at 3160 cm^−1^ confirmed that the phenolic –OH groups and hydrolysis product were introduced by the TA-APTES coating [33]. For the SP-TAPI, the broad peak between 3600 and 3100 cm^−1^ was assigned to the synergistic effects of the stretching vibration of –OH and –NH_2_ [34,35]. The peak located at 1664 cm^−1^ corresponded to the C=N stretching vibration, indicating that the in situ Schiff base reaction had occurred between the catechol groups of TA-APTES and the amino groups of the PEI [27]. More importantly, the peaks at 2950–2850 cm^−1^ were derived from the alkyl chain in PEI and APTES during the hydrolysis procedure [25,30]. The FTIR results clearly demonstrated that the hydroxyl and amino-containing functional moieties were successfully introduced onto the SP surface by a co-deposition modification, thereby significantly increasing the number of binding sites on the obtained SP-TAPI.

Additionally, the surface elemental composition of the SP-TAPI was analyzed by XPS (Table 1, Figure 1b). As shown in Figure 1b, the major chemical elements of the pristine SP were carbon and oxygen, while the N 1s, Si 2s, and Si 2p peaks were clearly identified in the XPS spectrum of the modified hybrids. Compared to the pristine SP, a significant increase in the N 1s content was observed in the XPS spectra of SP-(TA-APTES) and SP-TAPI, which was due to the introduction of nitrogen-containing functional groups during the co-deposition process [28]. The O/C ratio decreased from 0.43 to 0.23 because of the high content of carbon and low content of oxygen in the PEI and TA [31,36]. The overall data confirmed the covalent interaction between TA-APTES and PEI. The deconvolution of the C 1s spectra (Figure 1c) was curve-fitted into three or four main component peaks at the binding energies. There were three peaks (C–C, C–O, and C=O) in the C 1s spectra of the pristine SP. The appearance of the nitrous carbons (C–N) in the SP-(TA-APTES) was ascribed to the APTES because it did not occur in the pristine SP [27]. It was found that the peak belonging to the oxygenated carbons (C=O) exhibited a slight red-shift owing to the adjacent aromatic ring structure of the TA [34,35]. SP-TAPI exhibited a new C=N peak, which was ascribed to the chemical reaction product between the free pyrogallol moieties of TA-AP and the amino-containing PEI [25]; this result was consistent with the FTIR result. Therein, the XPS spectra also indicated the successful co-deposition of TA-APTES and PEI on the SP surfaces. To estimate the amount of PEI attached to the surface of the SP, TGA was conducted. As shown in Figure 1d, the amino-containing PEI molecules on the SP-TAPI composites comprised about 1.75 wt%. These results indicated that the amino groups were successfully attached to the backbone of the SP substrates through the two-step co-deposition procedure.

**Figure 2:**
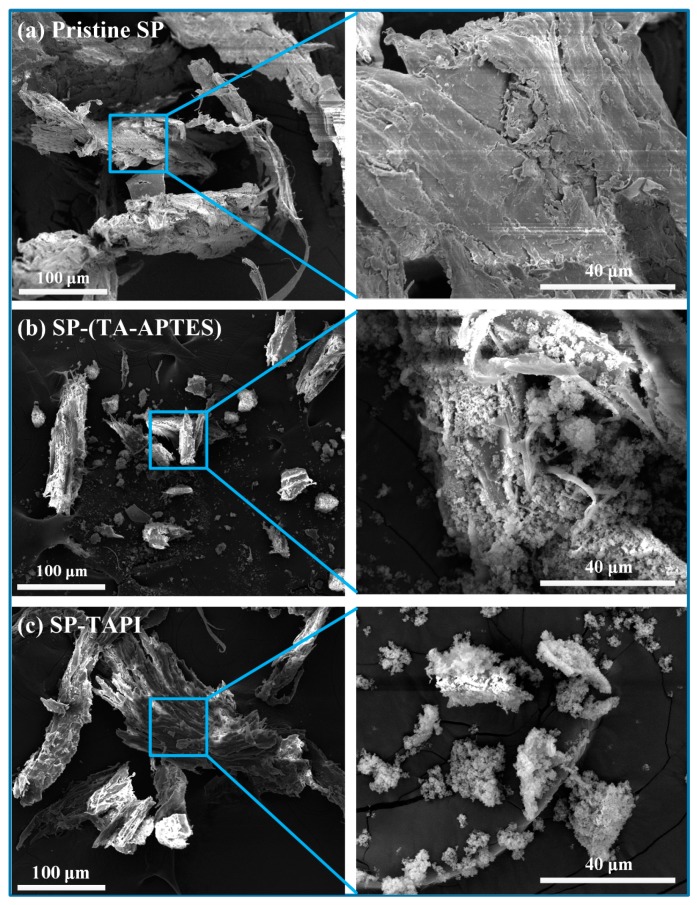
SEM images of (**a**) pristine SP, (**b**) SP-(TA-APTES), and (**c**) SP-TAPI composites, respectively.

**Figure 3:**
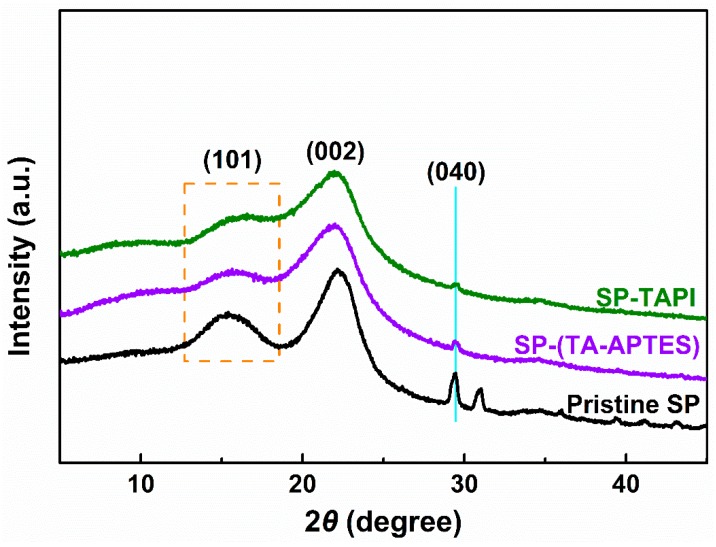
XRD patterns of pristine SP, SP-(TA-APTES), and SP-TAPI composites.

The microstructure of the SP and SP-TAPI composites was examined by SEM (Figure 2). The pristine SP had a random fibril-like structure and the surface was relatively smooth (Figure 2a). The fiber surface of the SP, modified by TA-APTES, was rough and a large number of particles were clearly observed on the sample surface (Figure 2b). After incubation in the PEI solution, a greater number of surface-bound particles were observed and they exhibited a typical hierarchical structure due to the introduction of PEI in copolymerization (Figure 2c) [28]. As a result, the deposited coating increased the porosity, thereby further enhancing the adsorption capacity of the as-obtained SP-TAPI composites [36]. XRD was used to investigate the structural evolution of the TAPI coating on the SP-TAPI composites; the patterns are shown in Figure 3. The pristine SP exhibited two diffraction peaks around 2θ = 16.3° and 22.1°, corresponding to the (101) and (002) planes of the cellulose structure [37]. The peaks of the (101), (002), and (040) still existed in the SP-TAPI hybrids but were weaker than in the pristine SP, suggesting that the crystal structure of the wood fibers was not changed after coating by the TA-APTES and PEI layer. The microstructure results indicated that the SP-TAPI exhibited a porous hierarchical structure, which improved the adsorption performance.

### 3.2. Adsorption Properties of Cd(II) Ions

**Figure 4:**
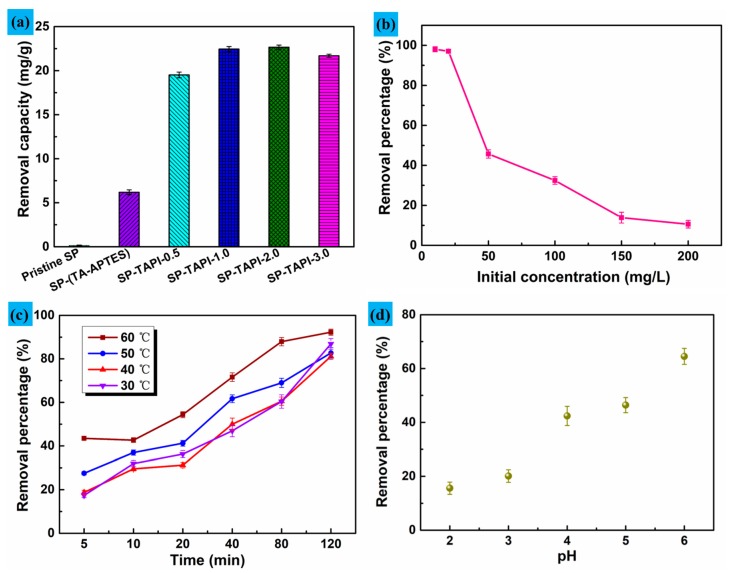
(**a**)The removal efficiency of Cd(II) of the pristine SP, SP-(TA-APTES), and SP-TAPI hybrids for different PEI amounts (Cd(II) initial concentration = 50 mg/L, pH = 5.0, m/V = 2, contact time = 3 h). (**b**) Effect of initial Cd(II) concentration on the Cd(II) removal efficiency by SP-TAPI (pH = 5.0, m/V = 10, contact time = 3 h). (**c**) Effect of contact time on the Cd(II) removal efficiency by SP-TAPI (Cd(II) initial concentration = 50 mg/L, pH = 5.0, m/V = 10). (**d**) Effect of pH on the Cd(II) removal efficiency by SP-TAPI (Cd(II) initial concentration = 50 mg/L, m/V = 10, contact time = 3 h).

The wood waste-derived SP-TAPI prepared under optimal conditions was used to treat the Cd(II)-containing wastewater. Considering the practical application of the adsorption process onto the wood fiber-based sorbents [12,20], the evaluation of all sorbents was based on the SP substrates (not involving PEI only). Thus, the effect of the different sorbents on the adsorption efficiency of Cd(II) was evaluated (Figure 4a); ~22.66 mg/g of removal capacity was achieved for the SP-TAPI-2.0, which was nearly 156-fold and 3-fold that of the pristine SP and SP-(TA-APTES), respectively. This removal capacity was much higher than that of most reported lignocellulose-based materials (as illustrated in Table 2). The improved Cd(II) removal capability of the SP-TAPI composite was attributed to a sufficient number of active adsorption groups on the hierarchical structure-endowed surfaces.

Figure 4b depicts the effects of initial Cd(II) concentrations on Cd(II) adsorption. The removal percentage of Cd(II) decreased gradually from the initial concentration of 50 mg/L to 200 mg/L, mainly owing to the limited number of available adsorption sites for a fixed adsorbent dosage at high initial Cd(II) concentrations [40,41]. During the adsorption process, the catechol and amino groups on the SP-TAPI composites might be responsible for Cd(II) adsorption. High Cd(II) concentrations caused a lack of affinity between bonding sites and absorbates, thereby diminishing their availability for adsorption.

The temperature plays an important role in the adsorption processes. The effects of temperature on the removal efficiency of Cd(II) were examined (Figure 4c). The removal percentage increased with increasing temperature, indicating that the increase in temperature promoted Cd(II) adsorption [42]. Therefore, the Cd(II) adsorption onto the SP-TAPI composites was an endothermic process.

It is known that the pH of the reaction system is a crucial factor during the adsorption process of metal ions because it influences the ionic state and species of the sorbents. Figure 4c shows that the Cd(II) removal percentage exhibited an increasing trend as the pH increased from 2.0 to 6.0, indicating that the Cd(II) adsorption onto SP-TAPI was dependent on pH. This may be attributed to the surface properties of the amino groups on SP-TAPI. When the pH was low, protonation of the –NH– group resulted in the easy formation of –NH^+^ cations, which were unfavorable for the adsorption of the positively charged Cd(II) ions by electrostatic attraction. With increasing pH, the amino groups of the adsorbent surface were gradually deprotonated, leading to an increase in –NH– sites on the surface of SP-TAPI [43,44]. In other words, the chelation ability with Cd(II) was enhanced significantly. The free phenolic hydroxyls and the −NH⋯OH− structure that formed on the SP-TAPI under higher pH conditions thus generated a strong electrostatic attraction with Cd(II) in the reaction system [7]. Crucially, this demonstrated that the Cd(II) adsorption capacity significantly decreased at pH ≥7.0, resulting from the formation of metal-hydrolyzed species such as soluble Cd(OH)+ and colloidal precipitate Cd (OH)_2_, to restrain complex formation [44,45]. Herein, the influence of the pH on the Cd(II) removal percentage further confirmed that electrostatic attraction played an important role in the adsorption process.

### 3.3. Analysis of the Interaction Mechanism

**Figure 5:**
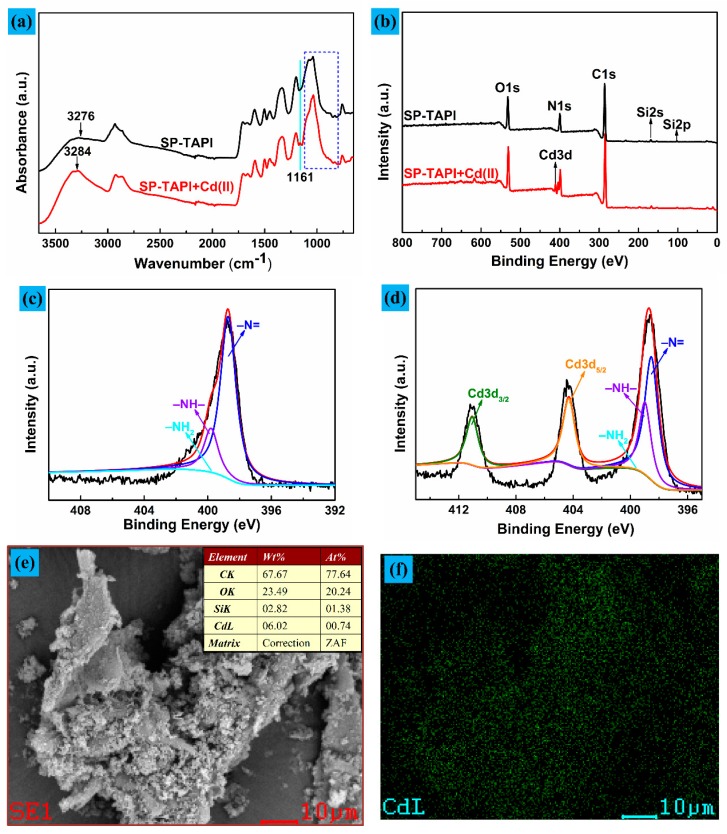
(**a**) FTIR and (**b**) XPS spectra of SP-TAPI before and after Cd(II) adsorption. (**c**) N 1s and (**d**) Cd 3d XPS spectra of the SP-TAPI composites before and after Cd(II) adsorption. (**e**) SEM image and (**f**) EDS spectrum of SP-TAPI after Cd(II) adsorption.

**Figure 6:**
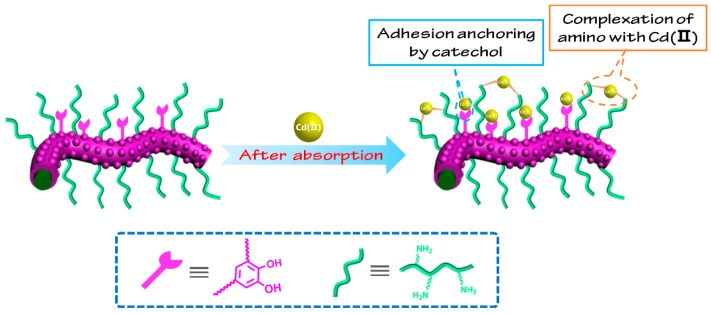
Schematic illustration of the proposed adsorption mechanism of Cd(II) ions by the SP-TAPI composites.

For an additional investigation of the possible potential adsorption sites and bonding modes, FTIR and XPS were used to detect the synthesized SP-TAPI after Cd(II) adsorption. As shown in Figure 5a, a wide peak relating to the –OH and –NH– vibration was observed; it shifted from 3276 to 3284 cm^−1^ after Cd(II) absorption, suggesting a strong interaction of the free phenolic hydroxyl and amino groups with the Cd(II) ions [5,40]. In addition, the intensity of the peaks in the low-frequency region slightly increased after Cd(II) absorption, which was likely due to the hybrid nitrogen-containing functional moieties on the SP-TAPI with Cd(II) [11]. Figure 5b illustrates the XPS wide-scan spectra of the SP-TAPI composite before and after Cd(II) adsorption. A distinct peak appeared after adsorption at a binding energy of approximately 405.1 eV and was assigned to Cd(II) [6]. As illustrated in Figure 5c, the magnification of the N 1s spectrum of the SP-TAPI could be divided into three components located at 399.0, 400.3, and 402.6 eV, corresponding to –NH=, –NH–, and –NH_2_, respectively [25]. After the adsorption of Cd(II), these peaks remained and the new binding energy peaks at 404.9 and 411.6 were assigned to Cd 3d_3/2_ and Cd 3d_5/2_, respectively (Figure 5d) [46], suggesting that the Cd(II) was removed entirely and was adsorbed on the SP-TAPI in the form of Cd(II). In addition, the composition of the composites after Cd(II) adsorption was verified by EDS (Figure 5e,f). The ratio of Cd was estimated at about 0.74% and uniform dispersion of Cd(II) was observed on the surface. These results indicate that the interaction mechanism of Cd(II) with the SP-TAPI composites was governed by electrostatic attraction and surface chelation (as illustrated in Figure 6).

## 4. Conclusions

In summary, green lignocellulose-based SP-TAPI hierarchical composites were prepared via a cost-effective method for the efficient adsorption of Cd(II). The surface chemistry, morphology, and interaction mechanism were investigated to evaluate the structural properties of the resulting SP-TAPI composites. The results indicated that the well-defined hierarchical structured sorbent had significant advantages over pristine wood waste. Owing to the versatile surface properties and distinct hierarchical structure of the (TA-APTES)/PEI hybrid layer, the removal capacity of Cd(II) by SP-TAPI was as high as 22.66 mg/g at pH = 5.0, which was higher than that of many reported lignocellulose-based materials. The pH had a significant effect on the adsorption process and the adsorption process was endothermic and spontaneous in nature. The findings in this study not only provide a cost-effective approach to prepare high-performance adsorbents but also greatly extend the efficient utilization of agricultural waste.

## Figures and Tables

**Table 1:** Surface element composition of the pristine SP substrate and modified SP-TAPI.

Sample	Composition (at %)				Atom Ratio	
	C	O	N	Si	O/C	N/C
Pristine SP	69.60	29.64	/	/	0.43	/
SP-(TA-APTES)	70.00	22.98	2.62	4.12	0.34	0.07
SP-TAPI	69.68	15.91	13.87	0.54	0.23	0.21

**Table 2:** Maximum absorption capacity (mg/g) reported in studies of the use of agricultural waste or lignocellulose-based materials.

Adsorbent	Adsorption Capacity (mg/g)	Ref
Sawdust (*Picea smithiana*)	2.87	[16]
Base-treated juniper fiber	29.5	[38]
Cellulose powder	17.2	[39]
Modified beech wood	14	[20]
Sugarcane bagasse and corn cob	15.5	[13]
